# Is Altered Oculomotor Control during Smooth Pursuit Neck Torsion Test Related to Subjective Visual Complaints in Patients with Neck Pain Disorders?

**DOI:** 10.3390/ijerph19073788

**Published:** 2022-03-23

**Authors:** Ziva Majcen Rosker, Miha Vodicar, Eythor Kristjansson

**Affiliations:** 1 Faculty of Sport, University of Ljubljana, 1000 Ljubljana, Slovenia; 2 Department of Orthopaedic Surgery, University Medical Centre Ljubljana, 1000 Ljubljana, Slovenia; miha.vodicar@kclj.si; 3 Landspitali University Hospital, 101 Reykjavik, Iceland; eythork@simnet.is

**Keywords:** visual disturbances, neck pain, oculomotor functions, eye movements

## Abstract

Subjective visual complaints are commonly reported in patients with neck pain, but their relation to objectively measured oculomotor functions during smooth pursuit neck torsion tests (SPNTs) has not yet been investigated. The aim of the study was to analyse classification accuracy of visual symptom intensity and frequency based on SPNT results. Forty-three patients with neck pain were referred by orthopaedic outpatient clinics where they were required to fill out 16-item proformas of visual complaints. Infrared video-oculography was used to measure smooth pursuit eye movements during neutral and neck torsion positions. Parameters of gain and SPNT difference (SPNT_diff_) were taken into the Naïve Bayes model as classifiers, while intensity and frequency of visual symptoms were taken as predicted class. Intensity and, to a lesser degree, frequency of visual symptoms previously associated with neck pain or focal vision disorders (computer vision syndrome) showed better classification accuracy using gain at neck torsion position, indicating cervical driven visual disturbances. Moreover, SPNT_diff_ presented with slightly lower classification accuracy as compared to gain at neck torsion position. Our study confirmed the relationship between cervical driven oculomotor deficits and some visual complaints (concentrating to read, words moving on page, blurred vision, difficulty judging distance, sore eyes, heavy eyes, red eyes, and eyes strain).

## 1. Introduction

The cervical spine has undergone scientific scrutiny for many years. From university professors to highly skilled clinicians, many attempts have been made to concur about interconnected signs and symptoms, but the overall understanding remains based on the assumptions that “In the cervical spine, everything is possible”. Many unfortunate people, therefore, suffer from a variety of different symptoms attributed to neck pain disorders [1,2]. While some relationship exists between functional signs and symptoms, such as in cervicogenic headaches [3], radiculopathy [4] and dizziness [5], visual disturbances remain poorly understood.

Visual disturbances have been reported over the years in those with neck pain disorders [5,6,7]. While some visual complaints, such as blurred vision, words jumping on the page, difficulty concentrating to focus and read, are more notably reported in those with neck pain disorders then others (i.e., double vision). It wasn’t until 2014 that Treleaven and Takasaki [8] presented more a comprehensive analysis of visual disturbances reported by individuals with neck pain. Their study presented a 16-item proforma to determine visual symptoms of which the most prevalent and troublesome visual complaints were identified. Out of these 16 items, some are suggested to be associated with cervical spine driven deficits [2,9] and some with focal vision disorders [10], whereas others that are not so commonly observed in patients with neck pain disorders are associated with vestibular pathology, ambient vision, and neurological disorders [11,12].

While visual symptoms associated with neck pain have received some attention in the literature [8], focal vision disorders and their relation to neck pain have been seldomly investigated. Focal vision disorder, also called computer vision syndrome, could co-exist in those with neck pain disorders. Moreover, computer vision syndrome can lead to occurrences of neck pain, and neck pain can contribute to the development of computer vision syndrome [13]. On the contrary, some other symptoms, such as double vision, could be a red flag condition found in patients with neck pain disorders (i.e., vertebral artery disfunction). To date, it is unknown which of the visual complaints and to what extent they are attributed to cervicogenic driven oculomotor deficits.

Oculomotor control of which the ability to smoothly follow a moving target with one’s eyes has been frequently investigated in clinical and research practice [6] and is altered in patients with neck pain disorders [5]. This is especially evident when the neck is torsioned to 45° to the left and to the right [14,15], called the smooth pursuit neck torsion test (SPNT) that has previously been identified as a reliable tool [16,17]. Neck pain patients frequently exhibit more sporadic saccadic jumps and lack the ability to focus their gaze on a moving target during the SPNT. Consequently, altered eye movement velocity as compared to target movement velocity (gain) is observed, especially in neck torsion position as compared to the neutral position expressed as the SPNT difference (SPNT_diff_) [18].

As to date, no well-established link has been identified between visual symptoms and oculomotor deficits in neck pain patients; it would be of clinical importance to assess such relationship. Visual disturbances remain subjective visual complaints that are often dismissed in clinical practice due to the lack of ability to identify concurrent gaze-related cervicogenic driven functional signs. Therefore, the aim of this study was to investigate whether oculomotor control during the SPNT is related to different visual symptoms commonly described by patients with neck pain disorders.

## 2. Materials and Methods

### 2.1. Participants

Patients with chronic neck pain were referred from orthopaedic outpatient clinics and were assessed for suitability via telephone interviews prior to participating in the study. All patients had to present with a minimum of 50° of cervical rotation to each side and be free from previous traumatic injury to the neck or head, shoulder or upper extremities pain, and any neurological or vestibular disorders, and were required to have not taken any medication or alcohol for the last 30 h prior to participating in the study. Prior to participation, they read and signed a consent form. The study was approved by the national medical ethics committee (number: 0120-47/2020/6) and was performed in accordance with the Declaration of Helsinki.

### 2.2. Assessment

Patients were required to mark pain intensity on a visual analogue scale (VAS). Each patient underwent a magnetic resonance imaging assessment prior to the initial screening at the orthopaedic outpatient clinic. This information was used to describe the extent and variability of cervical spine structural impairments. The SPNT protocol consisted of three different neck positions: (i) facing forward position (the trunk and head were in a neutral position), (ii) right neck torsion position at 45° (rotation of the trunk underneath the stationary head to 45° to the left), and (iii) left neck torsion position at 45° (rotation of the trunk underneath the stationary head to 45° to the right). Hip angle during sitting on a chair was 80° of flexion while feet were placed flat on the floor. All measurements were conducted by the same examiner in an isolated room with dimmed lights.

Before the test, all patients performed five familiarization warm-up cycles. For each condition patients were required to track 10 cycles of cyclic sinusoidal target movements with their eyes, followed by 1-min rest interval. Patients were tested at two different target movement profiles (40° of target movement amplitude with velocity of 30°s^−1^ and 30° of target movement amplitude with velocity of 30°s^−1^) at all three different neck positions. Different target movement velocities and amplitudes during all neck positions were performed in random order. After completing the SPNT at each neck position, recalibration of the eye-tracking device was performed during a 5-min rest.

Patients were required to complete a proforma of visual disturbances and complaints consisting of 16 items, as described by Treleaven and Takasaki [8]. The proforma included the following items: focal disorders (heavy eyes, sore eyes, red eyes, eye strain, visual fatigue, squinting, itchy eyes, and hard to focus on close work), vestibular pathology, migraines, ambient vision disturbances, or vertebral artery insufficiency (double vision, spots in eyes, sensitivity to light, and dizziness when reading) and symptoms associated with neck pain (blurred vision, words or objects moving, needing to concentrate to read, and difficulty judging distances). Patients were required to specify the average intensity of visual symptoms on a 3-level scale and average frequency on a 4-level scale. An examiner was present during the filling out of the proforma to ensure no items were left out unintentionally.

### 2.3. Equipment

**Figure 1:**
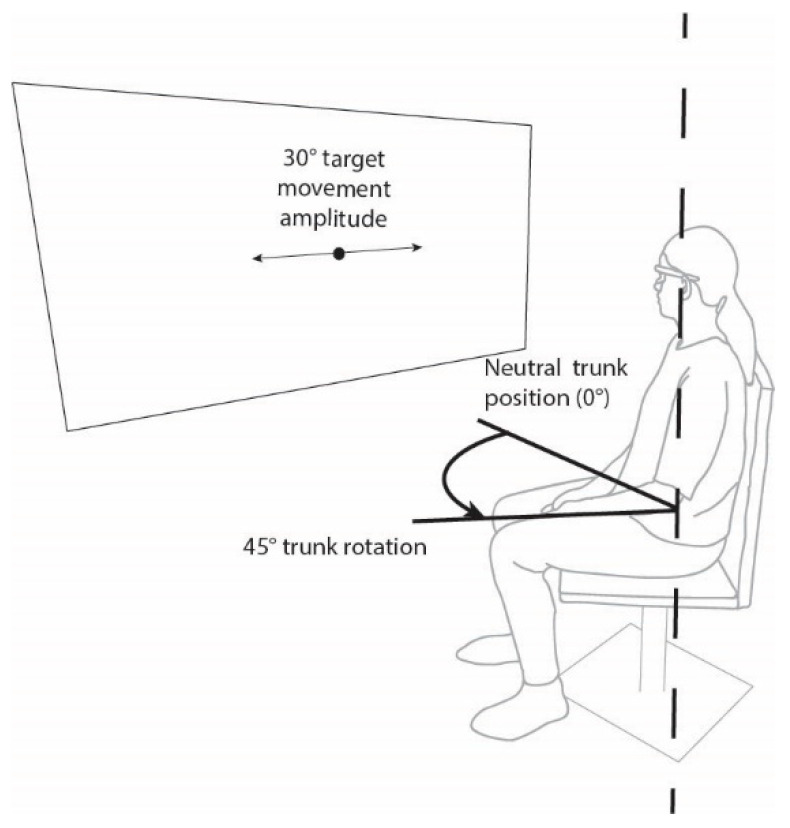
Patient setup.

Infrared video-oculography (Pro Glasses 2, Tobii, Danderyd, Sweden) was used to measure smooth pursuit eye movements at a sampling rate of 100 Hz [18,19,20]. A single target calibration routine was performed prior to measurements in the Tobii Pro Glasses Controller (Tobii Pro Glasses Controller, Tobii, Danderyd, Sweden). Patients were required to track a horizontally moving target of a red dot (size 0.5° of visual angle) projected (Optoma ML1050ST LED Projector, Fremont, CA, USA) on a white screen 150 cm away at an eye level with a 100-Hz refresh rate [21]. Patients were sitting on a custom-made rotatable chair with upper body fixed to the back support (Figure 1). A 16 item proforma was adapted as described by Teo et al. and Treleaven and Takasaki [8,13].

### 2.4. Data Analysis

The eye movement data were filtered for blinks and fixations using Tobii Pro Lab software (Tobii Pro lab 1.145, Tobii, Danderyd, Sweden). The square waves (saccades directed counter to each other and having an interval of relative standstill) and saccades were removed from the eye movement data using custom-written software in Matlab (R2017b, MathWorks, Natick, MA, USA). The eye movement data were fitted with a corresponding reference sinusoid with synchronized signal acquisition starting points. Each fitted reference sinusoid consisted of 10 cycles with correspondingly fixed amplitude (converted from angular degrees to pixels) and frequency that matched the profile for each individual condition. The first and last 10% of the amplitude in each eye movement cycle were removed from further analysis. The horizontal eye movements were analysed using gain, calculated as the ratio between eye velocity amplitude and visual target velocity amplitude, as described by Tjell et al. [18]. Gain torsion R represents the average gain during right neck torsion position from the 6th to 9th cycle, and gain torsion L represents the average gain during left neck torsion position from the 6th to 9th cycle [17]. In addition, smooth pursuit neck torsion difference (SPNT_diff_) was calculated, as presented in Equation (1). The calculation was adapted and is similar to that described by Tjell et al. [18]:

SPNT_diff_ = Gain neutral − (Gain torsion L + Gain torsion R)/2 (1)

Equation (1): gain neutral represents the average gain in the neutral position, gain torsion L represents the average gain during the left neck torsion position, and gain torsion R represents the average gain during the right neck torsion position.

For the 16-item proforma, the intensity of the visual symptoms was scaled as 1—mild, 2—moderate, 3—severe and for frequency as 1—rare, 2—occasional, 3—frequent, 4—always. If no visual symptoms were present, patients were instructed to leave the specific item blank (treated as zero score).

### 2.5. Statistical Analysis

Median and interquartile range were calculated for intensity and frequency score for each visual symptom as well as the percentage of patients reporting presence of individual visual complaints. Classification analysis was performed in Orange data-mining software (Orange 3.26.0, Ljubljana, Slovenia). To analyse the accuracy of the classifying intensity or frequency of visual disturbances using gain or SPNT_diff_, the Naïve Bayes machine-learning approach was used [22,23]. Gain and SPNT_diff_ at the most reliable and specific target movement profiles were used as predictor variables [24,25] and the score for intensity or frequency of each visual disturbance symptom as predicted class.

To develop the machine-learning classifier, data from 43 patients were randomly split into four folds. Three folds were used for model training and cross-validated with the remaining fold, repeating the procedure for all folds. Performance of the machine-learning classifier for each target movement profile was described by the area under the curve (AUC), sensitivity, and specificity.

## 3. Results

### 3.1. Participants

The mean age of 43 patients (31 females and 12 males) enrolled in this study was 41.3 ± 6.7 years (age range 25–51 years) with average pain duration of 13.4 ± 9.1 months and average VAS score 4.9 ± 1.7. The cervical spine magnetic-imaging assessment presented disc protrusions or herniations at the levels from C4 to Th1 in 27 patients, 9 patients presented with facet joints osteoarthritis at the levels from C5 to Th1, 12 patients presented with low-grade spondylolisthesis, and 9 patients presented with cervical spinal stenosis. Thirty-four patients presented with a combination of at least two types of structural deformities; however, in nine patients only one type of structural impairment was present.

Percentage of patients as well as median score with interquartile range for intensity and frequency of each visual symptom are presented in Table 1. As no patient reported presence of double vision, sensitivity to light, or spots in eyes, these symptoms were not included for further analysis. The highest percentage of patients reported presence of blurred vision and visual fatigue followed by sore eyes, heavy eyes, and other symptoms. Majority of reported visual symptoms showed mild (1) to moderate (2) intensity and rare (1) to frequent (3) frequency.

### 3.2. Classification Analysis

Results of the classification analysis are presented in Table 2. Classification accuracy (AUC) based on gain at neck torsion position proved to be higher for intensity as compared to frequency of visual symptoms. In addition, gain at neck torsion position proved to have higher classification accuracy as compared to gain at neutral position for both intensity and frequency of visual symptoms. The classification accuracy based on SPNT_diff_ proved to be slightly lower as compared to gain at neck torsion position for both intensity and frequency of visual symptoms. In general, classification accuracy was medium to low or non-present.

The highest classification accuracy of symptom intensity was observed for need to concentrate to read, followed by sore eyes, words moving on page, eye strain, heavy eyes, difficulty judging distance, blurred vision, and red eyes. A similar trend was observed for classifications using SPNT_diff_ but for a smaller number of visual symptoms for both intensity and frequency.

## 4. Discussion

The aim of this study was to investigate the relation between oculomotor control during the SPNT and different visual symptoms described by patients with neck pain disorders. Based on the results from our study, it can be concluded that intensity as opposed to frequency of visual symptoms is more related to the parameters of gain and SPNT_diff_, with both presenting with either moderate or low classification accuracy. Moreover, gain during neck torsion manoeuvre seems to have a more pronounced relation as compared to gain during neutral position, indicating that functional deficits of the cervical spine are related to some subjective visual complaints. SPNT_diff_ presented with slightly lower classification accuracy as compared to gain during neck torsion position. The highest classification accuracy based on gain was observed for the following visual symptoms: need to concentrate to read, sore eyes, words moving on page, eye strain, heavy eyes, difficulty judging distance, blurred vision, and red eyes. Similar trends were observed for SPNT_diff_, but fewer symptoms presented with at least low classification accuracy.

The results of our study confirm a stronger relationship between intensity of visual symptoms and SPNT performance than between frequency of visual symptoms and SPNT performance. This is in line with other studies reporting that intensity of subjective symptoms correlates to objective measures of neuromuscular functions of the neck in patients with neck pain disorders [26]. On the other hand, higher frequency of the symptoms would likely be present in those experiencing chronic symptoms. Chronic adaptations in those with spinal pain could lead to central nervous system adaptations that persist for a prolonged time even after alleviation of subjective symptoms of pain [27]. Such adaptations in the central nervous system could lead to alterations in oculomotor function that might be less dependent on symptomatic visual complaints. This notion is partially confirmed by studies reporting changes in oculomotor functions in asymptomatic subjects [28].

Functional connections between cervical spine deficits at neck torsion position and sensory mismatch commonly observed in neck pain patients could explain the relationship between gain at neck torsion position as well as SPNT_diff_ and certain visual complaints. These deficits might be related to errors in proprioceptive information derived from the neck, transmitted by the cervico-collic and cervico-ocular reflexes [14], which can only be observed if patients are actively holding their head while performing cyclic smooth pursuit eye movements. On the opposite these are likely not present when the head is supported by chin rest [29]. Neck muscles that possess high muscle spindle density can influence coordination of extraocular muscles and consequently oculomotor control [30]. In addition, proprioceptive information from extraocular muscles and sensory retinal information presents an important sensory source for detecting target movement direction and speed [29], where extraocular sensory feedback has been shown to be the primary source of information in environments with structured background [30,31]. The perception of target movement is an important driver for the control of smooth pursuit eye movements, which can be altered in patients with neck pain.

Symptoms that have previously been associated with neck pain disorders, concentrating to read, words moving on page, difficulty judging distance, and blurred vision [32,33] showed moderate relationships with gain at neck torsion angle and slightly less with SPNT_diff_. This information adds to findings reported by Gimse et al. [34] where moderate correlations were found between reading ability and SPNT_diff_. The presence of pain in the neck region would generally lead to stiffening of the neck muscles [35], which is accompanied by decreased eyes–head–shoulders coordination and consequently decreased ability to maintain focal vision on a target [36]. Increased focal vision oscillations would prevent steady-state gaze, which might result in an increased number of saccades. This could lead to blurred vision, which would demand higher concentration while reading or keeping the gaze on a moving target. The symptom, “words moving on page”, also called oscillopsia, can appear resulting from abnormal eye movements caused by upper cervical spine instability or by impaired vestibulo-ocular reflex [37] that can also be found in patients with neck pain disorders [38]. Difficulty judging distance is a common symptom reported by patients with neck pain disorders and can result from altered eye vergence. Our study found a relationship between difficulty judging distance and gain in neck torsion position. This is in accordance with other studies that found decreased vergence abilities when the neck was placed in torsion compared to the neutral position [39].

Prolonged time spent in the position where focal vision has to be frequently maintained could lead to focal vision disorders that are suggested to worsen with prolonged use of computers and mobile devices [10]; therefore, focal vision disorders are also called computer vision syndrome. Our study found a more pronounced relationship between gain during neck torsion position and some of the symptoms commonly associated with focal vision disorders (sore eyes, heavy eyes, red eyes, and eye strain), indicating the influence of cervical driven deficits on focal vision disturbances. The notion of the contribution of neck pain on symptomatic visual disturbances has also been confirmed by the study performed by Teo et al. [13]. Based on the results from their study, intensity of visual symptoms was more pronounced in the neck pain group than in the control group, regardless of similarities in daily computer use. Although growing evidence is emerging that musculoskeletal conditions, such as neck pain, might be related to computer vision syndrome, that relationship is poorly explained. It is currently still unknown whether computer vision syndrome causes neck pain or neck pain causes computer vision syndrome.

None of the patients from our study reported double vision or diplopia, sensitivity to light, and spots in eyes; therefore, these symptoms were not further analysed. Although Treleaven and Takasaki [8] reported on the possibilities that sensitivity to light and spots in eyes could be related to neck pain disorders, double vision should be considered as part of a differential diagnosis, such as vertebral artery insufficiency [40,41].

Important limitations of the study were that a heterogenous group of neck pain patients were included in the analysis, altogether possibly influencing the results of our study. Future studies should subgroup patients based on the level and region of pain [42] and traumatic and nontraumatic origin of neck pain disorders [43]. It could be expected that the relations between subjective visual complaints and the SPNT would be greater when investigating whiplash associated disorder patients due to the involvement of upper cervical spine trauma and its direct neurophysiological connection to the visual and vestibular systems [2]. In our study, only one aspect of oculomotor control was studied (continuous smooth pursuit eye movements) and did not address other connections between oculomotor control and subjective visual complaints in neck pain patients. Visual symptoms should therefore also be studied in relation to other oculomotor functions, such as smooth pursuit initiation, eye vergence, saccade accuracy, and other daily visual demanding tasks [44]. Another limitation of our study was that the amount of time individuals spent using computers or similar devices was not considered, which could have importantly influenced classification accuracy reported in our study. Therefore, future studies should gather this information for further analysis.

## 5. Conclusions

Our study confirmed the relationship between cervical driven oculomotor deficits measured during the SPNT and some of the commonly reported visual complaints in patients with neck pain disorders. Intensity of visual symptoms should be considered in clinical practice as it might show a more pronounced relationship to oculomotor control deficits measured during neck torsion positions. Although some relationship was found between visual complaints and oculomotor deficits related to cervical spine, other potential causes not investigated in our study should be considered.

## Figures and Tables

**Table 1:** Median, interquartile range, and percentage of patients reporting presence of each individual visual complaint.

	Intensity ^1^		Frequency ^2^		
Visual Symptom	Median ^3^	IQR ^4^	Median ^3^	IQR ^4^	% of Patients ^5^
Concentrating to read	2	2	2	2	63.6%
Words moving on page	0	2	0	3	45.5%
Blurred vision	1	1.5	3	2	81.8%
Difficulty judging distance	2	2	2	2.5	63.6%
Sore eyes	1	1.5	3	3.5	72.7%
Heavy eyes	2	2.5	2	3	72.7%
Harder to do close work	1	2	2	2.5	54.5%
Visual fatigue	2	1	3	1.5	81.8%
Itchy eyes	1	1	1	2.5	63.6%
Red eyes	1	1.5	2	3	63.6%
Eye strain	1	1.5	2	4	63.6%
Squinting	1	2	2	3	54.5%

^1^ Intensity—intensity score for an individual visual symptom; ^2^ Frequency—frequency score for an individual visual symptom; ^3^ Median—median score for individual visual symptom; ^4^ IQR—interquartile range for individual visual symptom; ^5^ % of patients—percentage of patients reporting individual visual symptom.

**Table 2:** Relation between visual symptoms, gain, and SPNT_diff_.

		Gain Torsion ^1^			Gain Neutral ^2^			SPNT_diff_^3^		
	Visual Symptom	AUC ^4^	Se ^5^	Sp ^6^	AUC ^4^	Se ^5^	Sp ^6^	AUC ^4^	Se ^5^	Sp ^6^
Intensity	Dizzy reading	0.340	0.282	0.438	0.296	0.388	0.339	0.440	0.482	0.638
	Concentrating to read	0.734	0.763	0.814	0.513	0.614	0.716	0.634	0.563	0.814
	Words moving on page	0.652	0.746	0.753	0.489	0.475	0.361	0.638	0.746	0.853
	Blurred vision	0.545	0.464	0.758	0.427	0.352	0.583	0.515	0.444	0.708
	Difficulty judging distance	0.578	0.611	0.893	0.353	0.407	0.693	0.498	0.611	0.893
	Sore eyes	0.704	0.667	0.909	0.525	0.375	0.667	0.644	0.667	0.809
	Heavy eyes	0.600	0.589	0.873	0.534	0.450	0.714	0.586	0.539	0.873
	Harder to do close work	0.493	0.328	0.838	0.389	0.448	0.762	0.481	0.310	0.638
	Visual fatigue	0.477	0.417	0.600	0.279	0.483	0.500	0.462	0.411	0.602
	Itchy eyes	0.297	0.225	0.683	0.348	0.319	0.702	0.255	0.264	0.483
	Red eyes	0.539	0.473	0.836	0.366	0.329	0.604	0.515	0.273	0.833
	Eye strain	0.629	0.518	0.867	0.431	0.455	0.758	0.525	0.618	0.859
	Squinting	0.366	0.436	0.606	0.415	0.401	0.723	0.301	0.236	0.703
Frequency	Dizzy reading	0.412	0.406	0.716	0.176	0.273	0.408	0.347	0.209	0.581
	Concentrating to read	0.626	0.718	0.640	0.486	0.519	0.572	0.614	0.518	0.713
	Words moving on page	0.536	0.520	0.687	0.361	0.242	0.400	0.622	0.608	0.748
	Blurred vision	0.682	0.606	0.833	0.466	0.145	0.681	0.693	0.515	0.815
	Difficulty judging distance	0.484	0.509	0.481	0.347	0.382	0.412	0.463	0.494	0.569
	Sore eyes	0.337	0.281	0.734	0.331	0.091	0.611	0.512	0.545	0.769
	Heavy eyes	0.433	0.373	0.538	0.308	0.182	0.749	0.376	0.242	0.675
	Harder to do close work	0.679	0.582	0.698	0.428	0.397	0.494	0.454	0.545	0.768
	Visual fatigue	0.572	0.573	0.644	0.473	0.432	0.532	0.472	0.114	0.651
	Itchy eyes	0.353	0.114	0.538	0.372	0.300	0.580	0.321	0.273	0.623
	Red eyes	0.357	0.327	0.645	0.480	0.361	0.544	0.357	0.364	0.722
	Eye strain	0.462	0.321	0.561	0.458	0.364	0.577	0.496	0.364	0.720
	Squinting	0.383	0.421	0.525	0.410	0.339	0.465	0.500	0.455	0.628

^1^ Gain torsion—gain measured at left and right neck torsion position with two target movement profiles (40° amplitude/30°s^−1^ velocity and 30° amplitude/30°s^−1^ velocity); ^2^ Gain neutral—gain measured at neutral position measured with two target movement profiles (40° amplitude/30°s^−1^ velocity and 30° amplitude/30°s^−1^ velocity); ^3^ SPNT_diff_—smooth pursuit neck torsion difference; ^4^ AUC—area under the receiver operating characteristic curve; ^5^ Se—true positive rate (sensitivity); ^6^ Sp—true negative rate (specificity).

## Data Availability

The data presented in this study are available on request from the corresponding author.
